# Viral infections as triggers in autoimmune blistering diseases: A systematic review of associations and mechanisms

**DOI:** 10.1016/j.jdin.2025.09.007

**Published:** 2025-10-03

**Authors:** Henry Tseng, John W. Frew, Dédée F. Murrell

**Affiliations:** a Department of Dermatology, Liverpool Hospital, Sydney, New South Wales, Australia; b School of Clinical Medicine, Faculty of Medicine and Health, UNSW Sydney, New South Wales, Australia; c Department of Dermatology, St. George Hospital, Sydney, New South Wales, Australia

**Keywords:** autoimmune blistering disease, bullous pemphigoid, COVID-19, environmental exposure, Epstein-Barr virus, hepatitis virus, herpes simplex virus, immunobullous disease, immunology, pemphigus, SARS-CoV-2, virus diseases

*To the Editor:* The role of environmental factors in autoimmune blistering disease (AIBD) pathogenesis remains unclear, though they appear to significantly influence disease onset and exacerbation.^1^ Growing evidence implicates infectious triggers in the pathogenesis of AIBDs, with viral agents—particularly herpesviruses—drawing increasing attention. We conducted a systematic review of studies investigating viral associations with AIBDs, including pemphigus vulgaris, pemphigus foliaceus, and bullous pemphigoid. Eligible studies (cross-sectional, case-control, or cohort) were identified through PubMed and Scopus from inception to April 2025 in accordance with the Preferred Reporting Items for Systematic Reviews and Meta-Analyses (PRISMA) guidelines, with 31 studies included after full-text screening (Fig 1). Case reports and studies without laboratory confirmation of viral infection were excluded.

**Fig 1:**
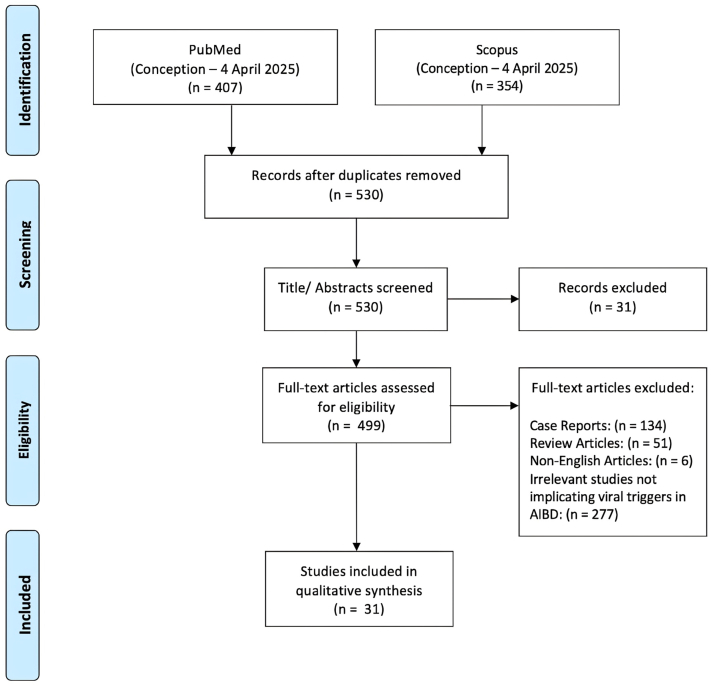
PRISMA flow diagram. *AIBD*, Autoimmune blistering disease; *PRISMA*, Preferred Reporting Items for Systematic Reviews and Meta-Analyses.

The most frequently implicated virus among all those evaluated was herpes simplex virus (HSV). Several studies revealed the presence of HSV DNA in lesional or peripheral samples of different forms of pemphigus. In one of the studies by Konda et al, following a 10-day course of acyclovir, HSV-positive PV patients showed clinical improvement, suggesting a possible therapeutic effect.^2^ However, contradictory serologic findings have been reported by others, and different sampling methodologies adopted in different studies (eg, swab vs biopsy) limited cross-study comparability. Several studies detected Epstein-Barr virus, cytomegalovirus, or human herpesvirus-6/7 with uncertain clinical significance and many without proper controls. Human herpesvirus 8 had stark geographic disparity—positive findings from China and Iran contrasted with consistently negative results from Europe and the United States—underscoring concerns about background prevalence and PCR specificity.^3^

SARS-CoV-2 has emerged as a possible immunologic trigger. In a population-based study, Curman et al reported increased AIBD incidence within 3 months of COVID-19 infection (hazard ratio: 1.51, 95% confidence interval: 1.26-1.81), while COVID-19 vaccination appeared protective (hazard ratio: 0.51, 95% confidence interval: 0.39-0.67).^4^ Proposed mechanisms include oxidative stress–induced desmoglein antibody production and IgG4 skewing.^4^ Although case reports were excluded, their temporal proximity to disease onset offers clinically meaningful signals. Hepatitis B virus was associated with AIBDs in one large Israeli cohort but not in smaller Turkish studies^5^; findings for hepatitis C virus were inconsistent.

A key challenge was differentiating viral causality from reactivation in immunosuppressed patients. Many studies depended on cross-sectional data and, thus, could not infer the temporal sequence of events. Further interpretation is hindered by having no standardized detection methods for viruses (eg, serology versus PCR) and inconsistencies in the sample types tested (eg, blood, swab, tissue). Notably, skin biopsy-based PCR may offer greater sensitivity in immunosuppressed populations, yet remains underutilized.

While causality remains unproven, the balance of existing evidence, particularly for HSV or SARS-CoV-2, suggests that viral infections may act as cofactors in AIBD development or flares. Future longitudinal studies with standardized virologic assessment in treatment-naïve patients are needed. Broader virome analyses may also uncover novel viral contributors, much like the identification of HHV-8 in Kaposi sarcoma.

## Conflicts of interest

None disclosed.
